# Supplementary figures and images for: Organoid modeling reveals the tumorigenic potential of the alveolar progenitor cell state
Source: EMBO J. 2025 Feb 10;44(6):1804–28. doi: 10.1038/s44318-025-00376-6 (PMC11914084; doi:10.1038/s44318-025-00376-6)

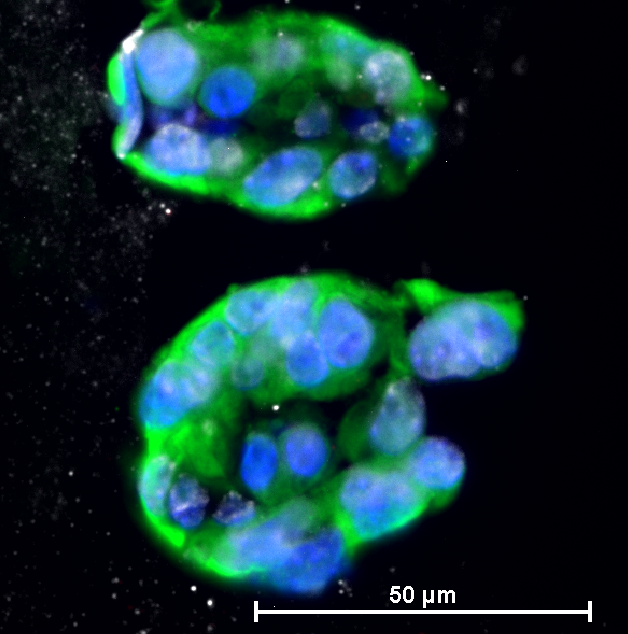

Supplement: Supplementary file 2 — Source data Fig. 3 [file 44318_2025_376_MOESM2_ESM.zip › 3F/Hmga2-high only all channel .tif]

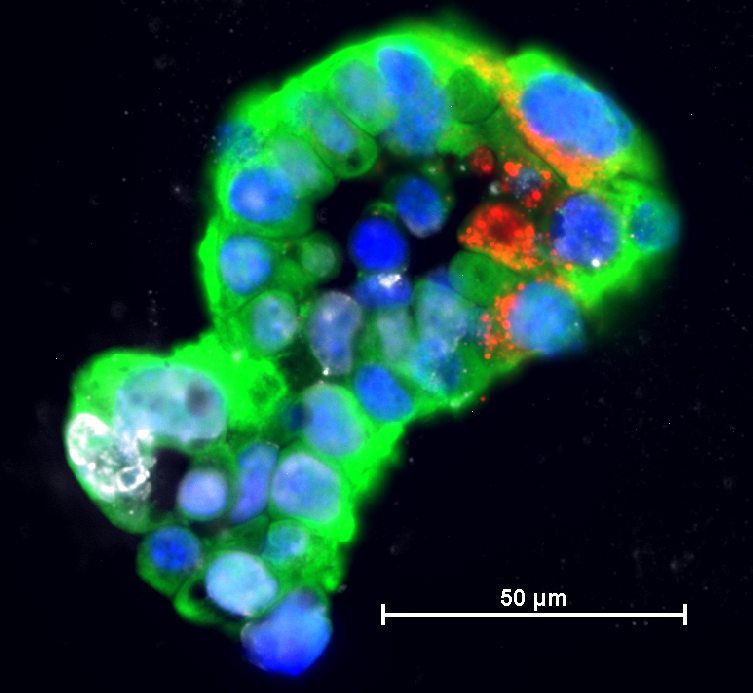

Supplement: Supplementary file 2 — Source data Fig. 3 [file 44318_2025_376_MOESM2_ESM.zip › 3F/Mixed all channel.tif]

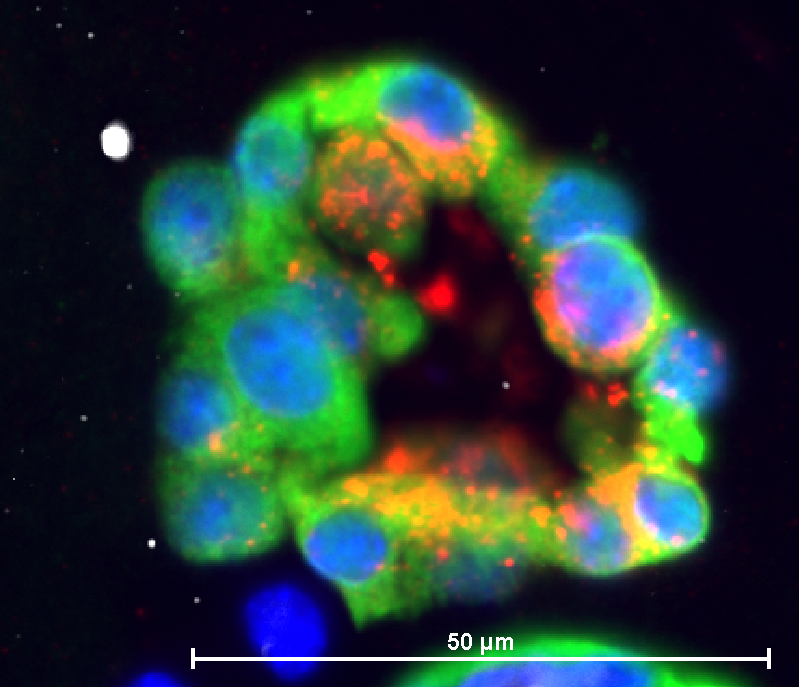

Supplement: Supplementary file 2 — Source data Fig. 3 [file 44318_2025_376_MOESM2_ESM.zip › 3F/SPC-high only all channel.tif]

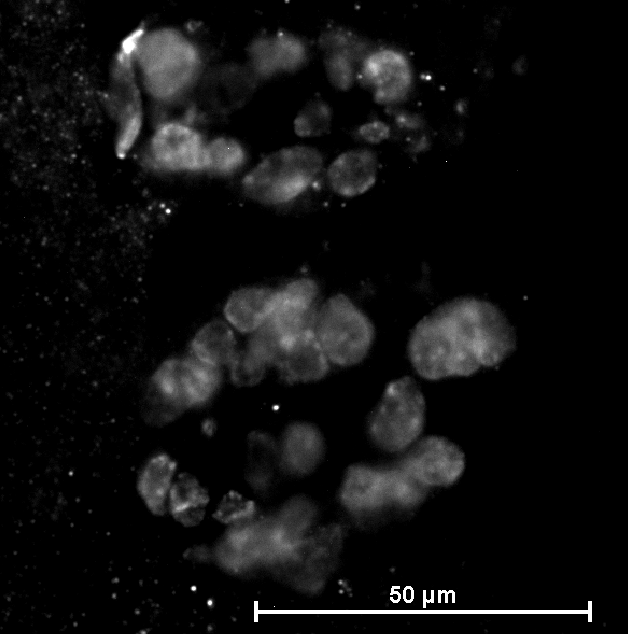

Supplement: Supplementary file 2 — Source data Fig. 3 [file 44318_2025_376_MOESM2_ESM.zip › 3F/Hmga2-high only Hmga2 channel.tif]

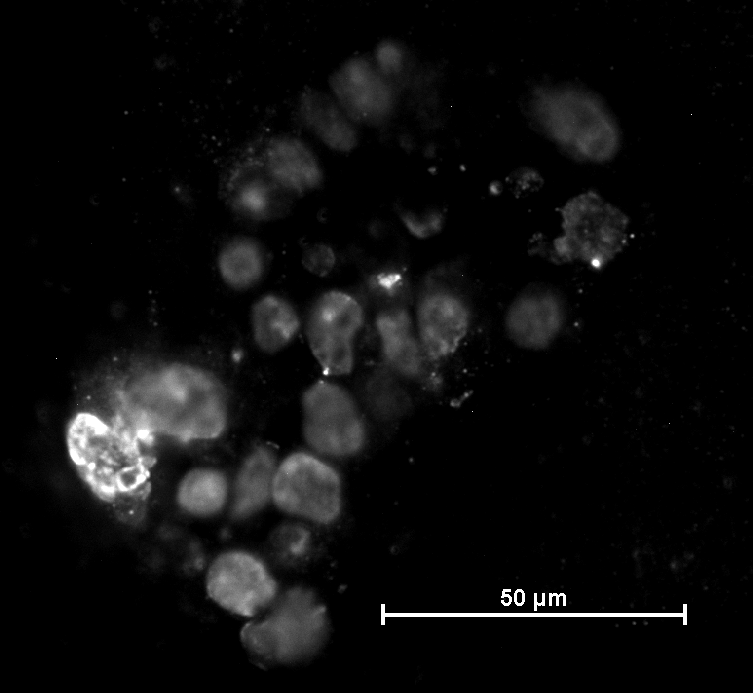

Supplement: Supplementary file 2 — Source data Fig. 3 [file 44318_2025_376_MOESM2_ESM.zip › 3F/Mixed Hmga2 channel.tif]

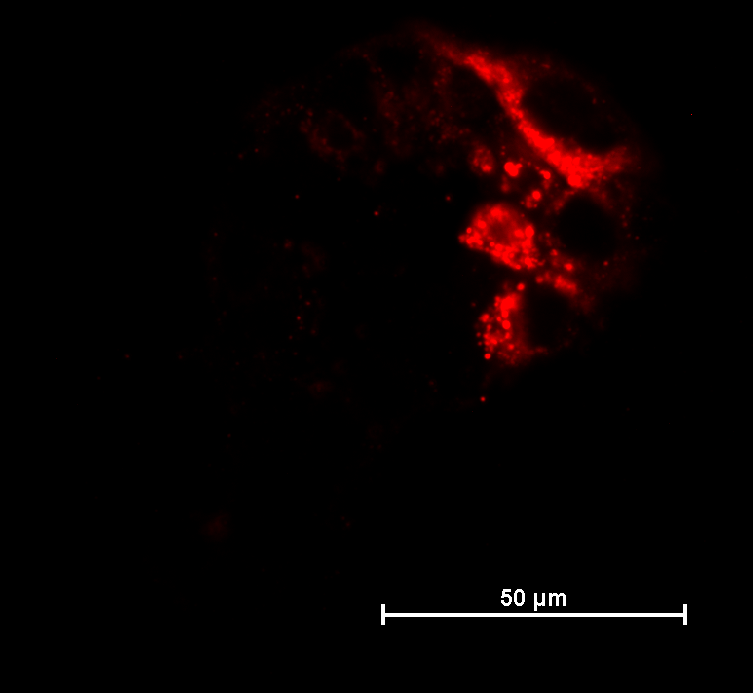

Supplement: Supplementary file 2 — Source data Fig. 3 [file 44318_2025_376_MOESM2_ESM.zip › 3F/Mixed SPC channel.tif]

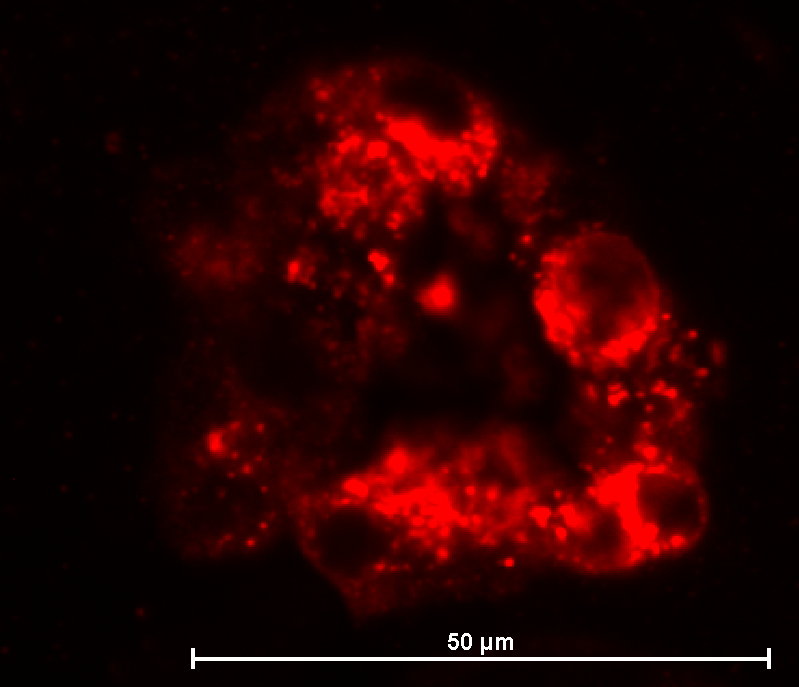

Supplement: Supplementary file 2 — Source data Fig. 3 [file 44318_2025_376_MOESM2_ESM.zip › 3F/SPC-high only SPC channel.tif]

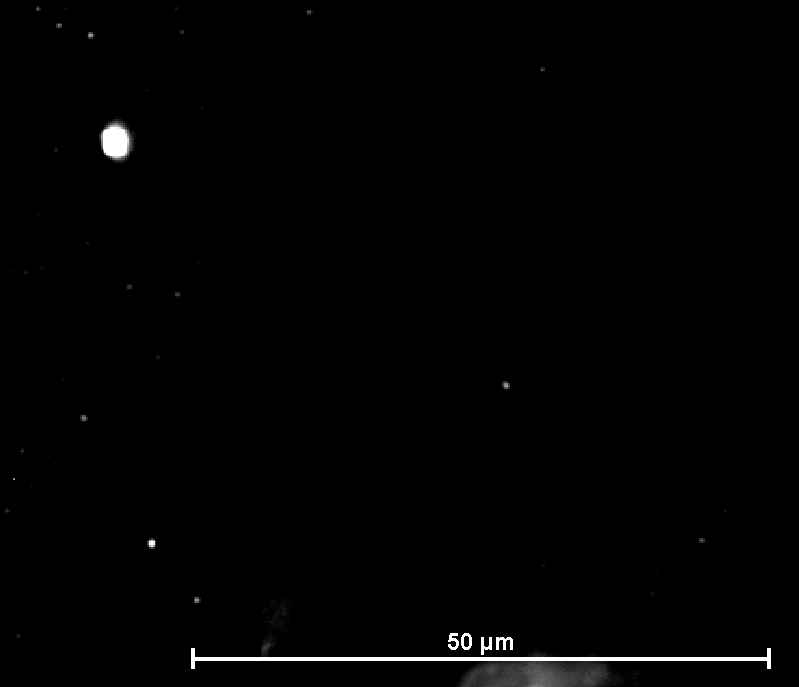

Supplement: Supplementary file 2 — Source data Fig. 3 [file 44318_2025_376_MOESM2_ESM.zip › 3F/SPC-high only Hmga2 channel.tif]

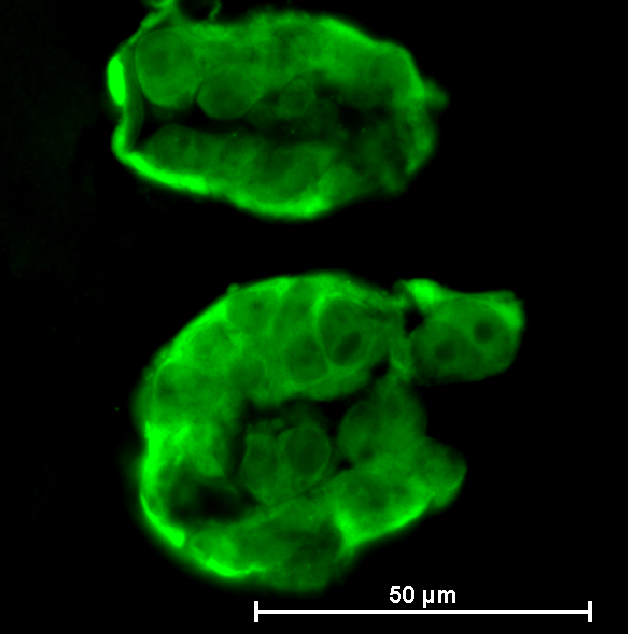

Supplement: Supplementary file 2 — Source data Fig. 3 [file 44318_2025_376_MOESM2_ESM.zip › 3F/Hmga2-high only GFP channel.tif]

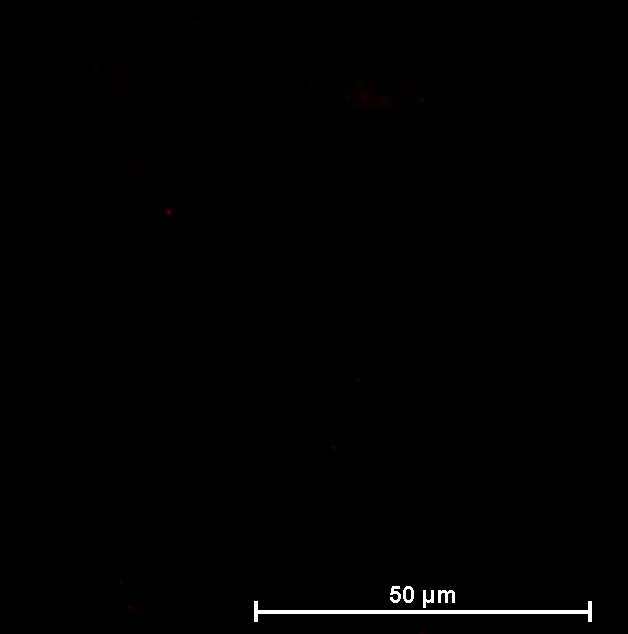

Supplement: Supplementary file 2 — Source data Fig. 3 [file 44318_2025_376_MOESM2_ESM.zip › 3F/Hmga2-high only SPC channel.tif]

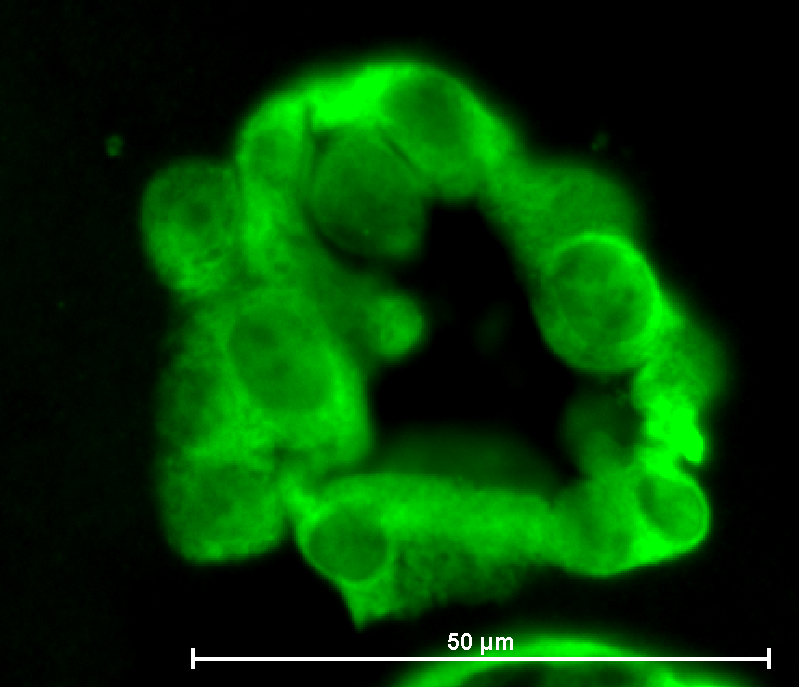

Supplement: Supplementary file 2 — Source data Fig. 3 [file 44318_2025_376_MOESM2_ESM.zip › 3F/SPC-high only GFP channel.tif]

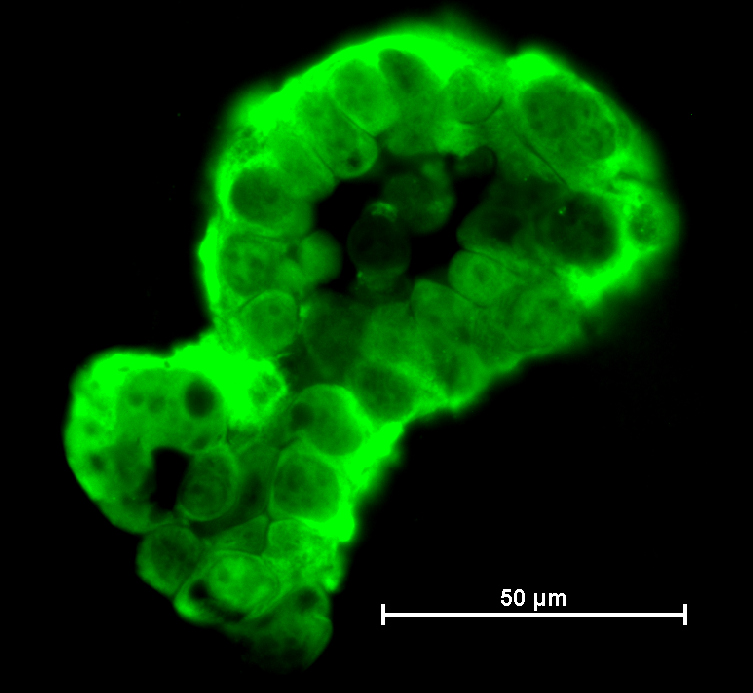

Supplement: Supplementary file 2 — Source data Fig. 3 [file 44318_2025_376_MOESM2_ESM.zip › 3F/Mixed GFP channel.tif]

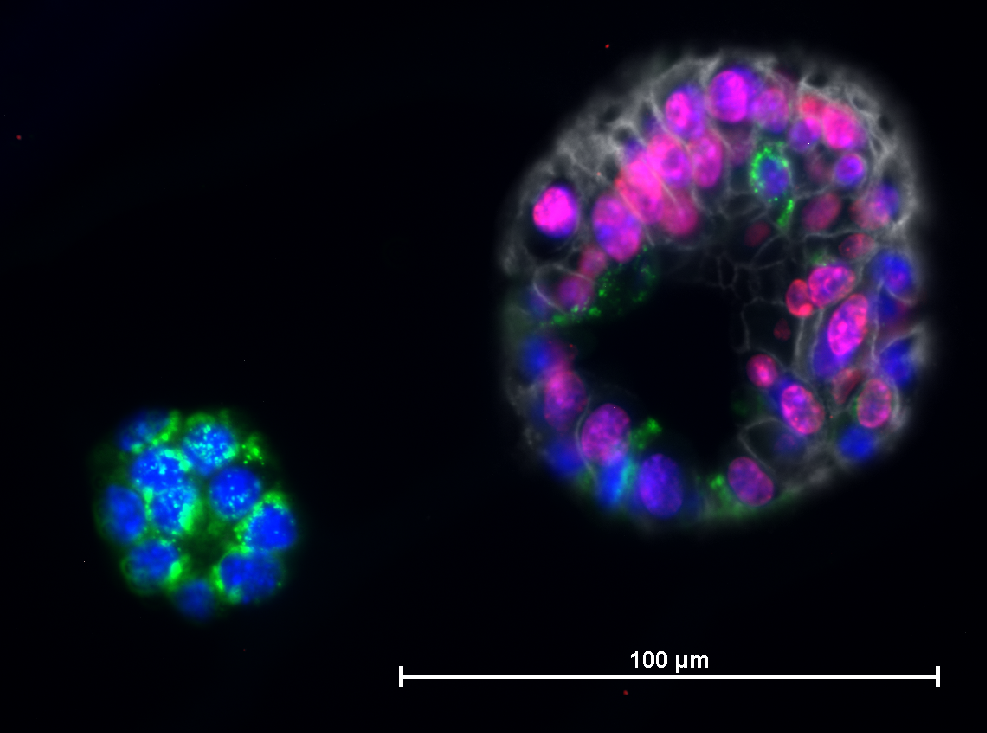

Supplement: Supplementary file 3 — Source data Fig. 4 [file 44318_2025_376_MOESM3_ESM.zip › 4C/all.tif]

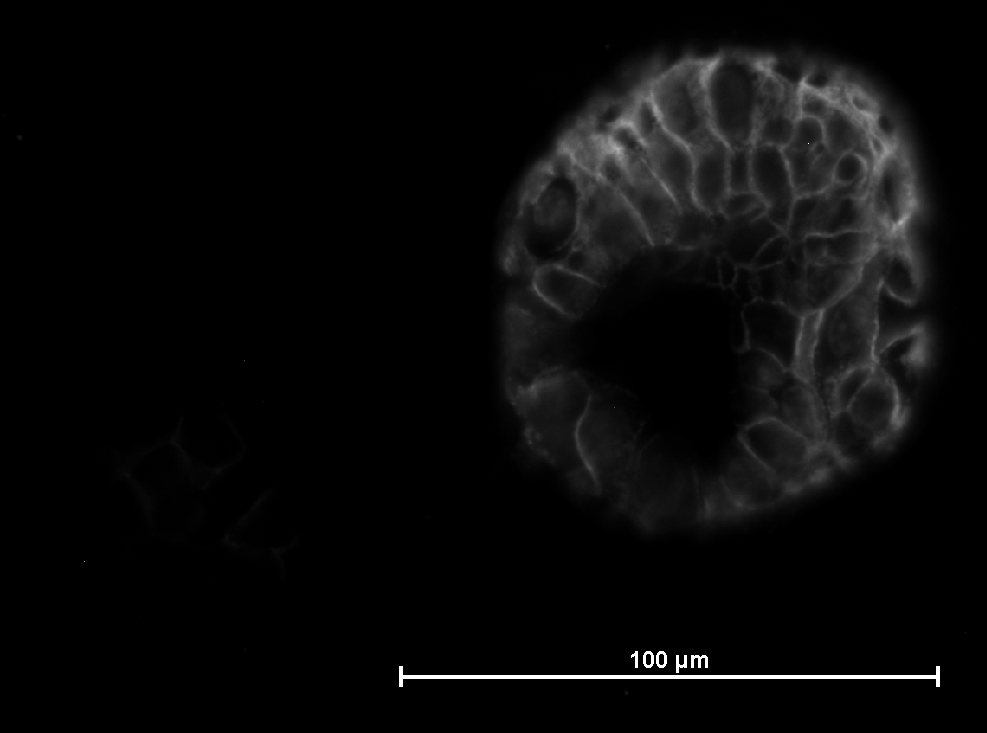

Supplement: Supplementary file 3 — Source data Fig. 4 [file 44318_2025_376_MOESM3_ESM.zip › 4C/CD44.tif]

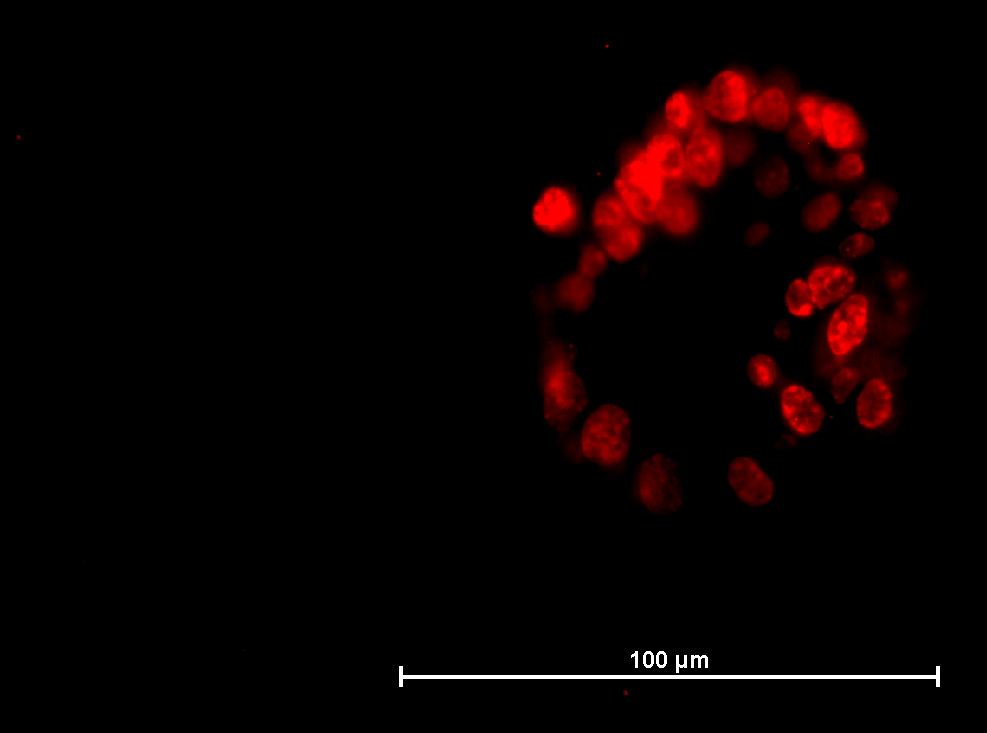

Supplement: Supplementary file 3 — Source data Fig. 4 [file 44318_2025_376_MOESM3_ESM.zip › 4C/Hmga2.tif]

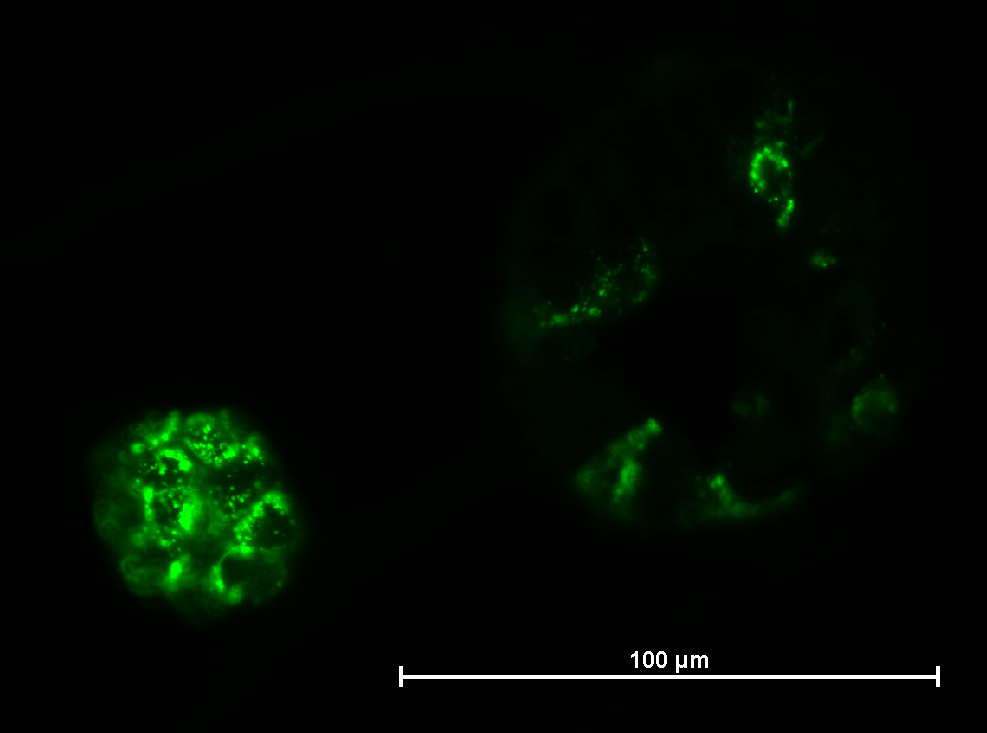

Supplement: Supplementary file 3 — Source data Fig. 4 [file 44318_2025_376_MOESM3_ESM.zip › 4C/SPC.tif]

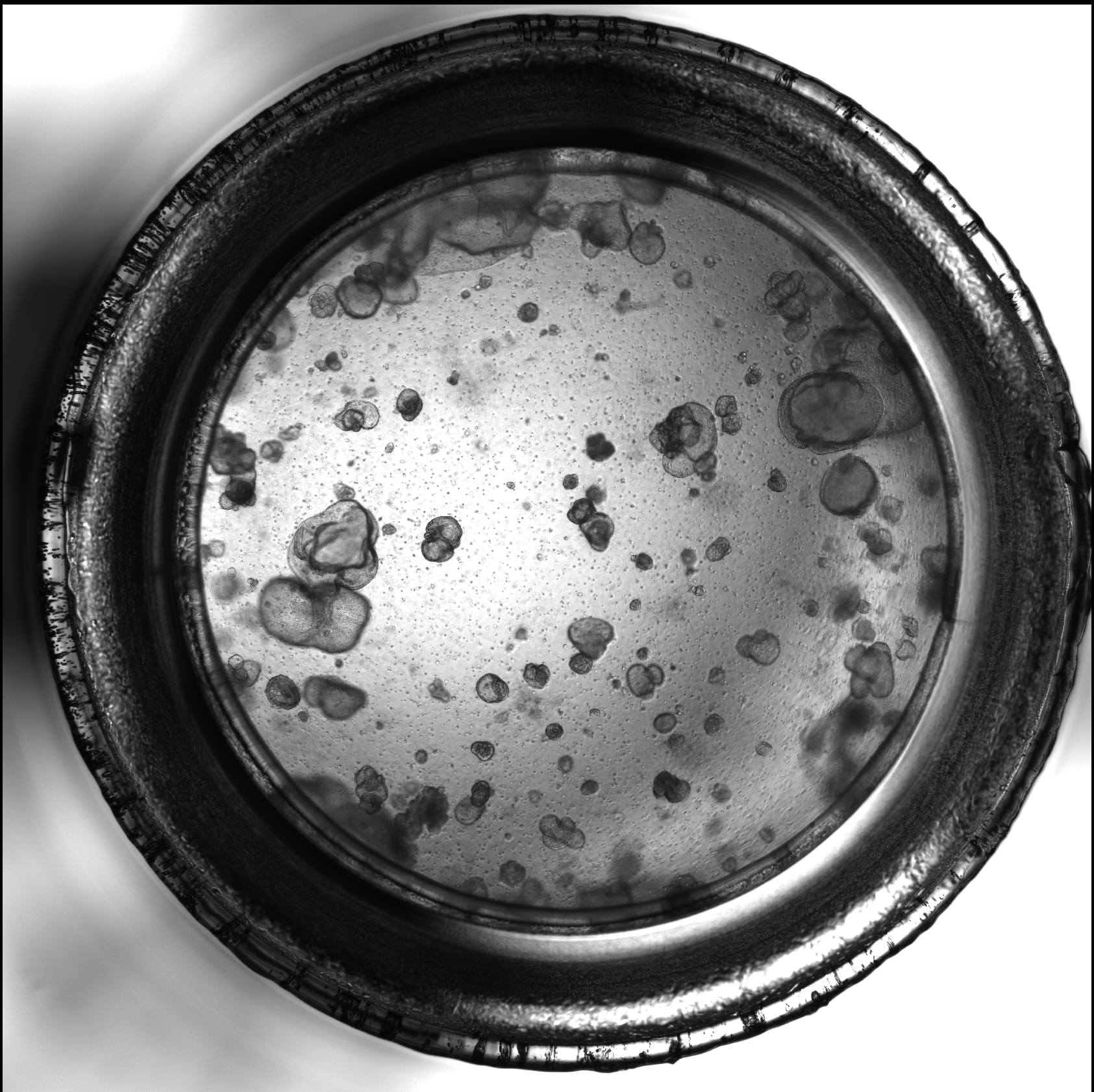

Supplement: Supplementary file 3 — Source data Fig. 4 [file 44318_2025_376_MOESM3_ESM.zip › 4F/cd44 low co-culture rep2.tif]

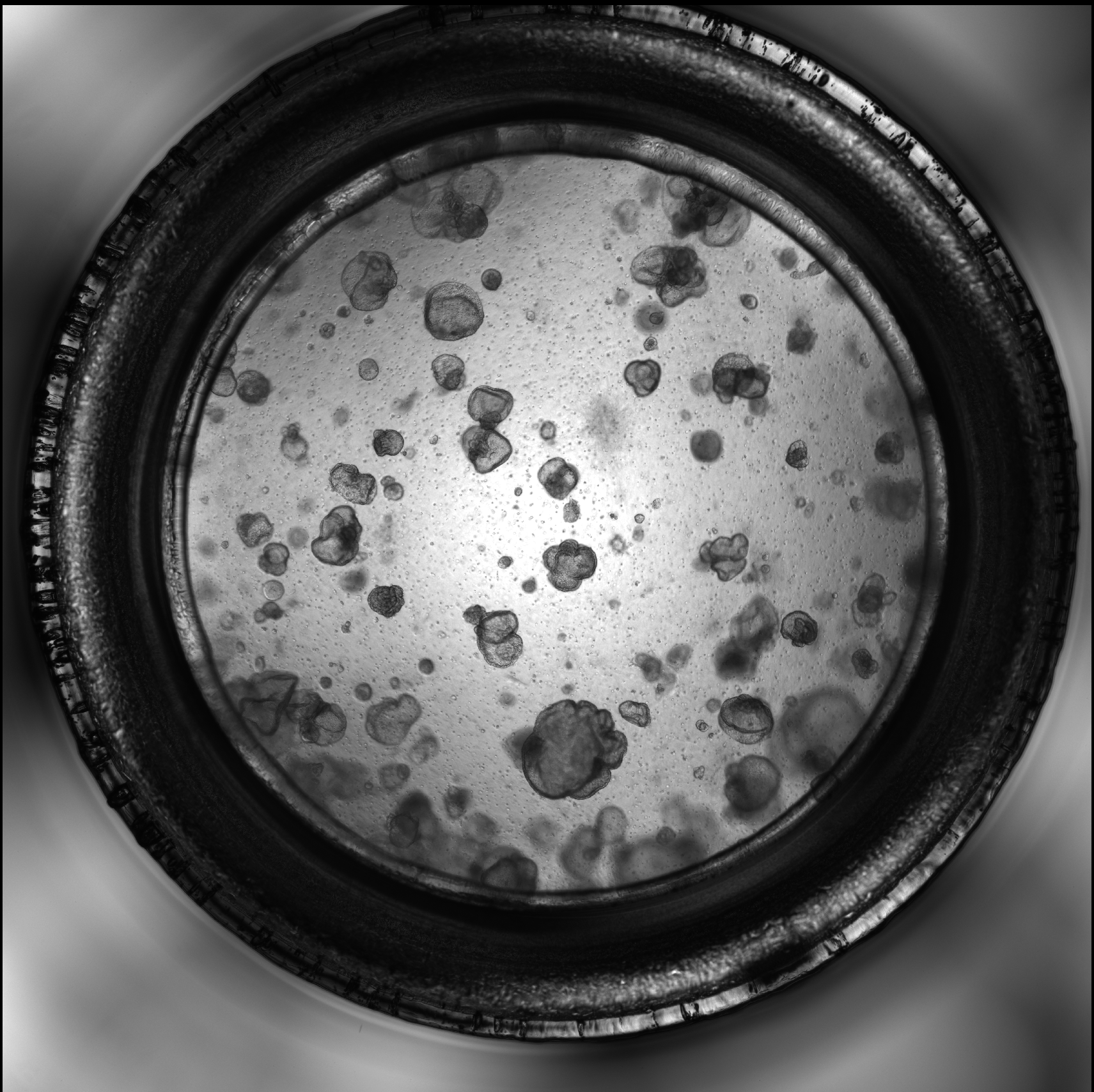

Supplement: Supplementary file 3 — Source data Fig. 4 [file 44318_2025_376_MOESM3_ESM.zip › 4F/CD44 low co-culture rep1.tif]

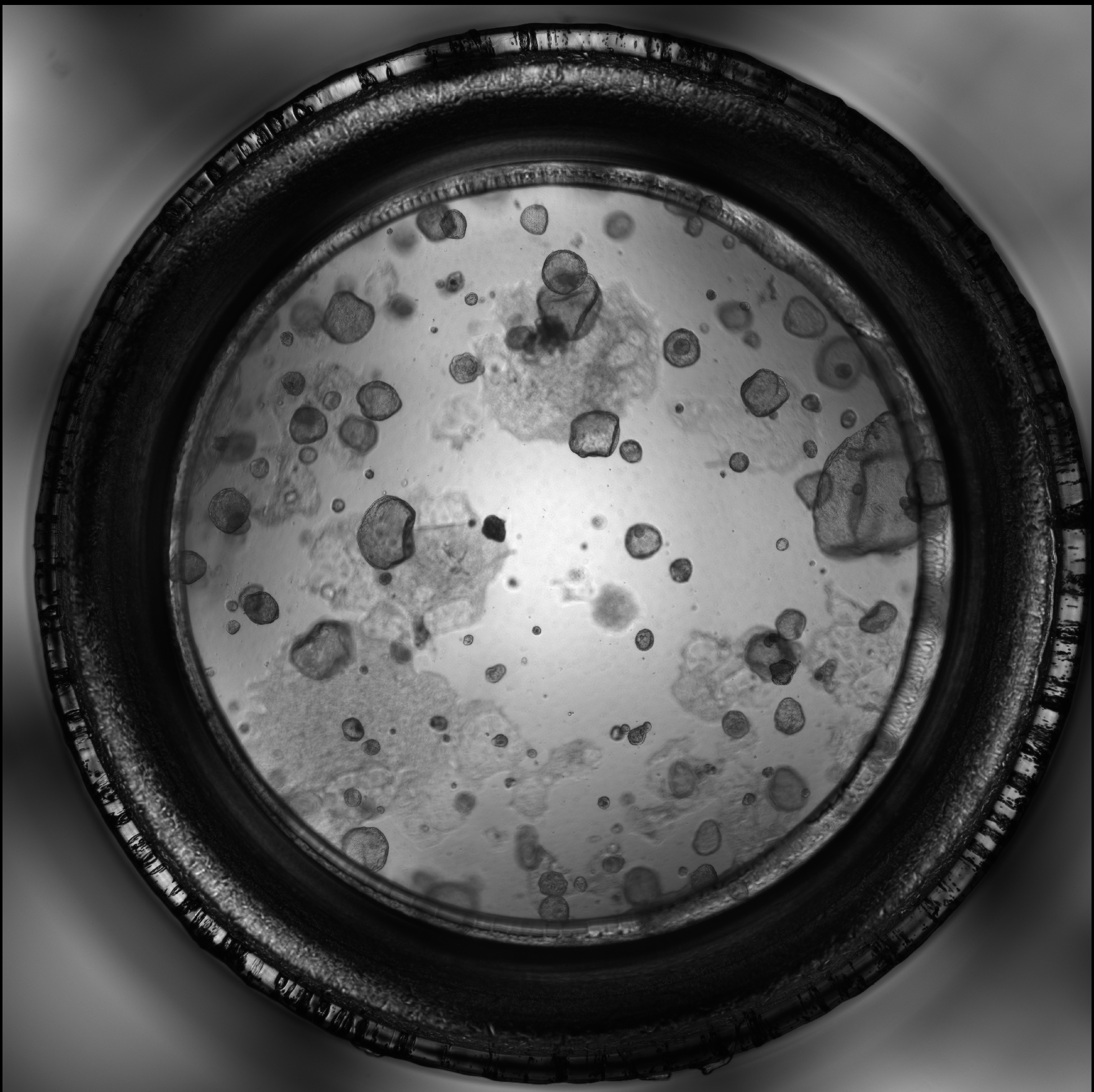

Supplement: Supplementary file 3 — Source data Fig. 4 [file 44318_2025_376_MOESM3_ESM.zip › 4F/cd44 high mono-culture rep2.tif]

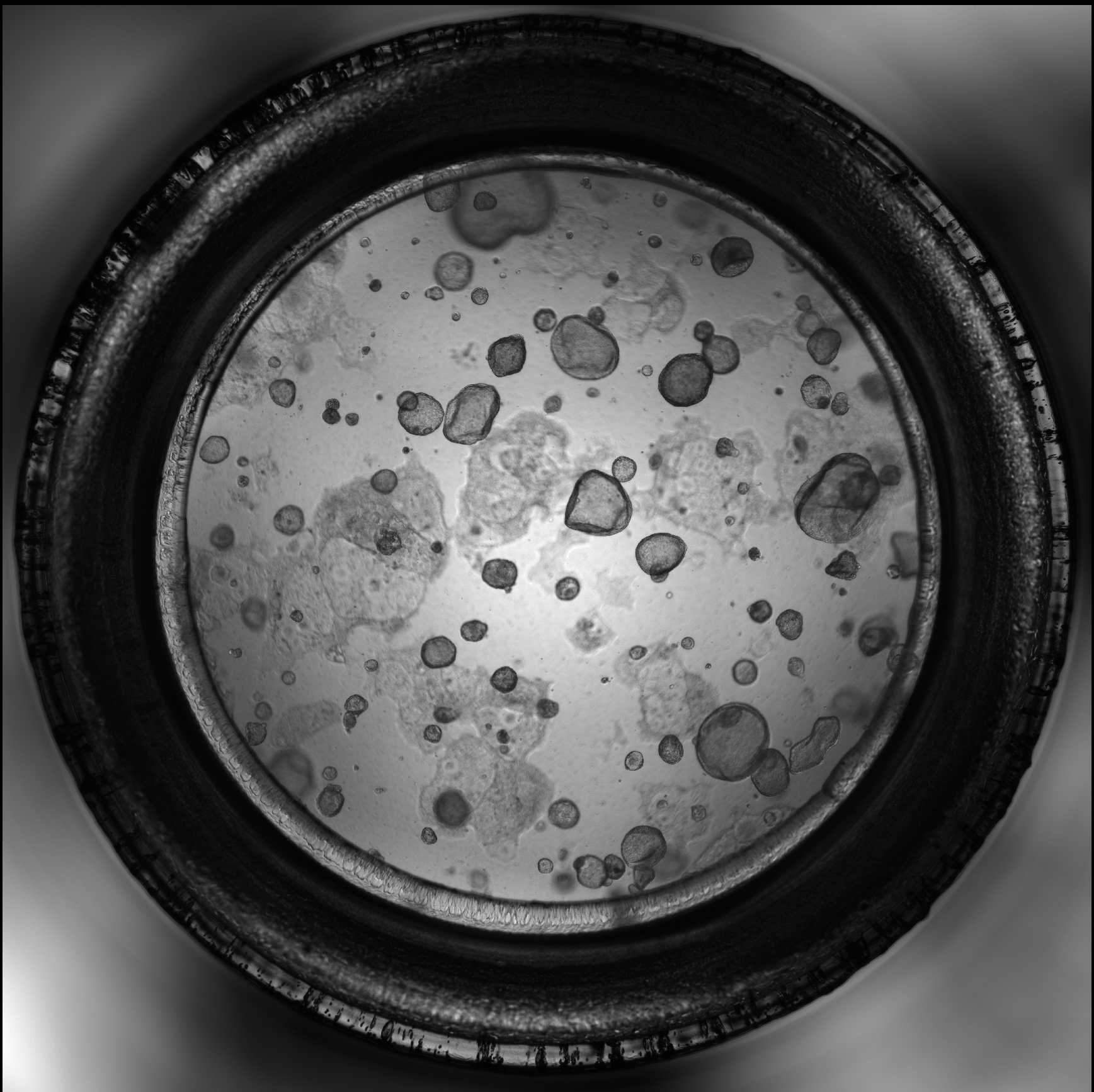

Supplement: Supplementary file 3 — Source data Fig. 4 [file 44318_2025_376_MOESM3_ESM.zip › 4F/cd44 high mono-culture rep1.tif]

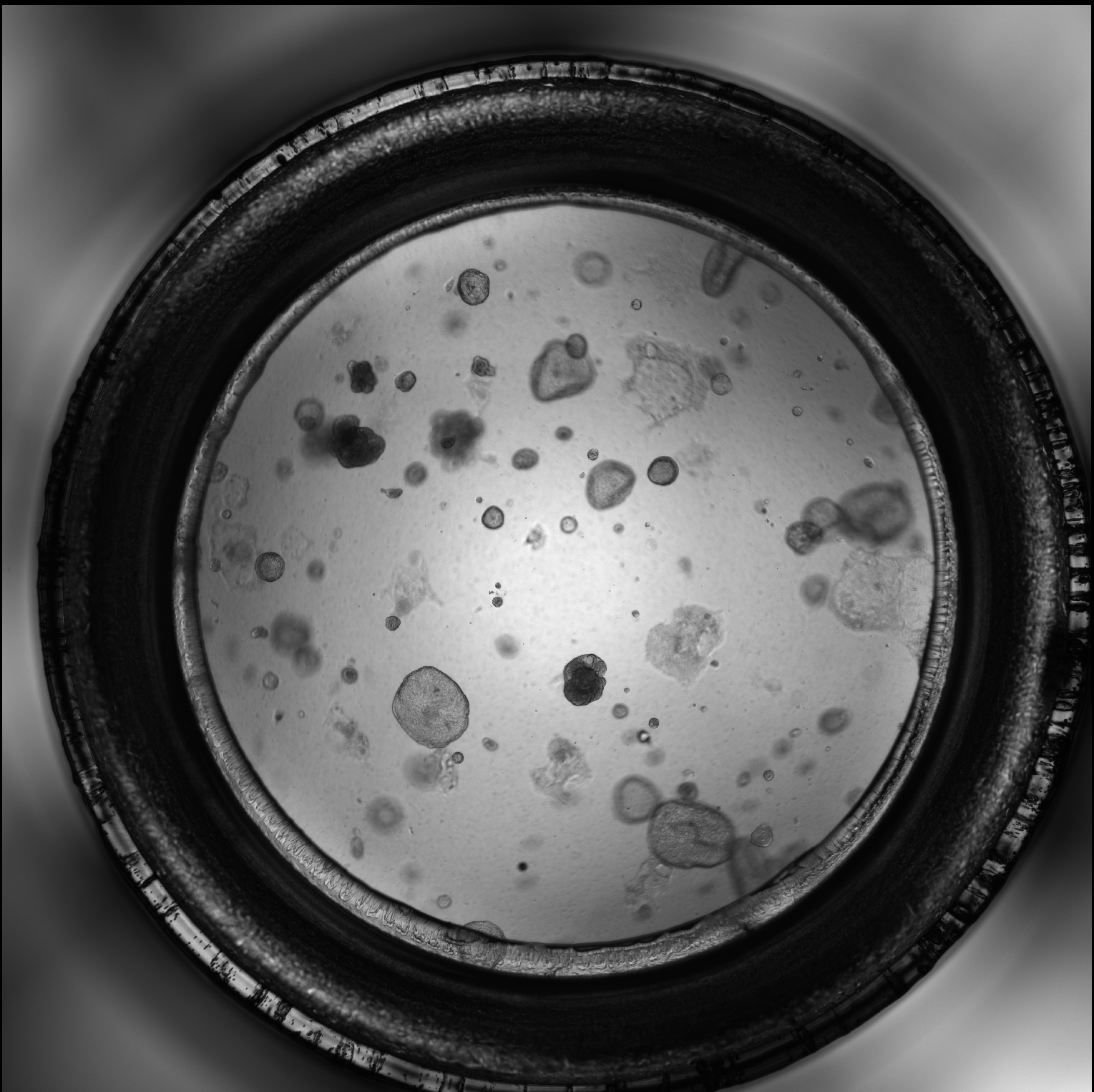

Supplement: Supplementary file 3 — Source data Fig. 4 [file 44318_2025_376_MOESM3_ESM.zip › 4F/cd44 low mono-culture rep1.tif]

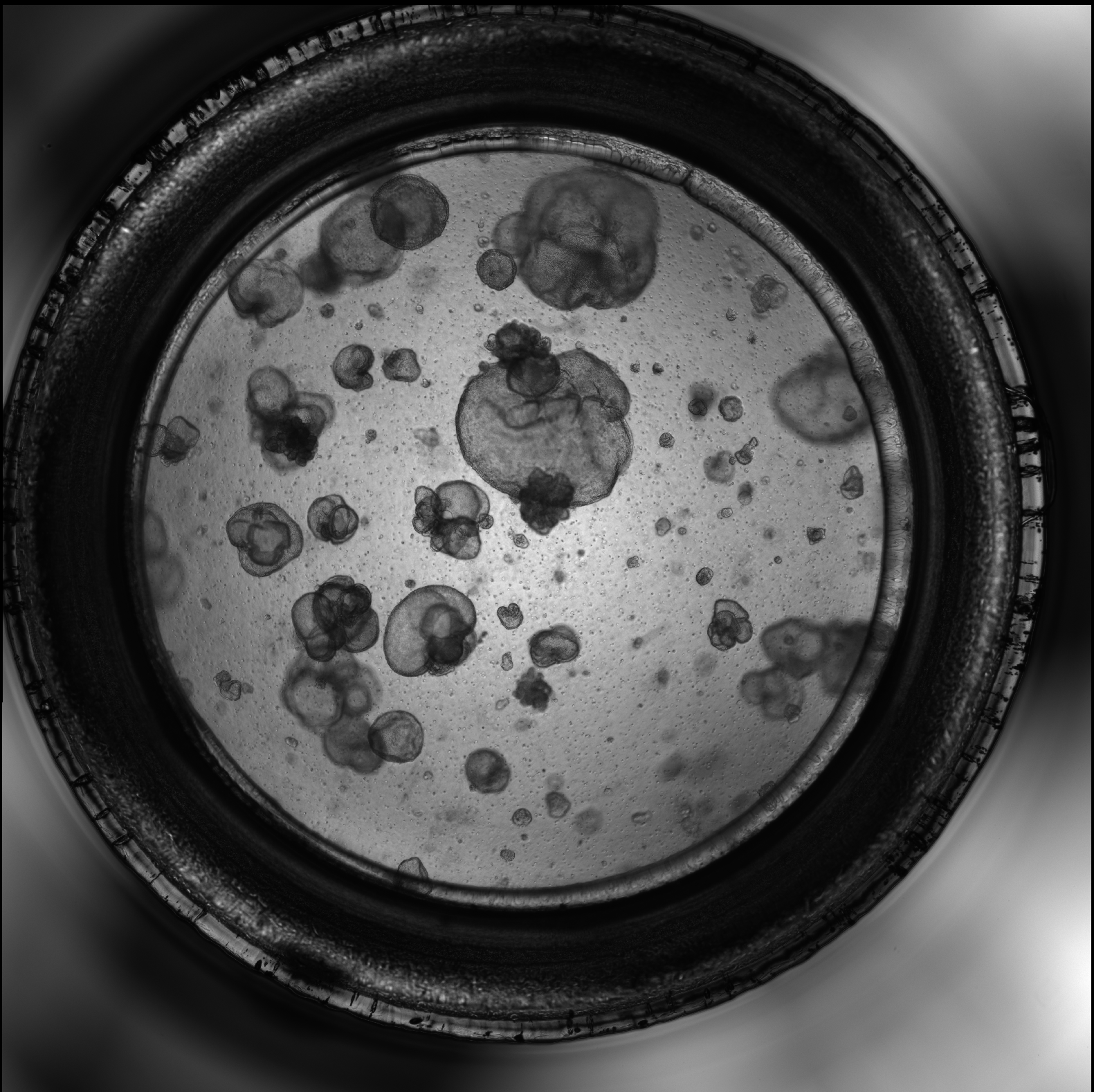

Supplement: Supplementary file 3 — Source data Fig. 4 [file 44318_2025_376_MOESM3_ESM.zip › 4F/cd44 high co-culture rep2.tif]

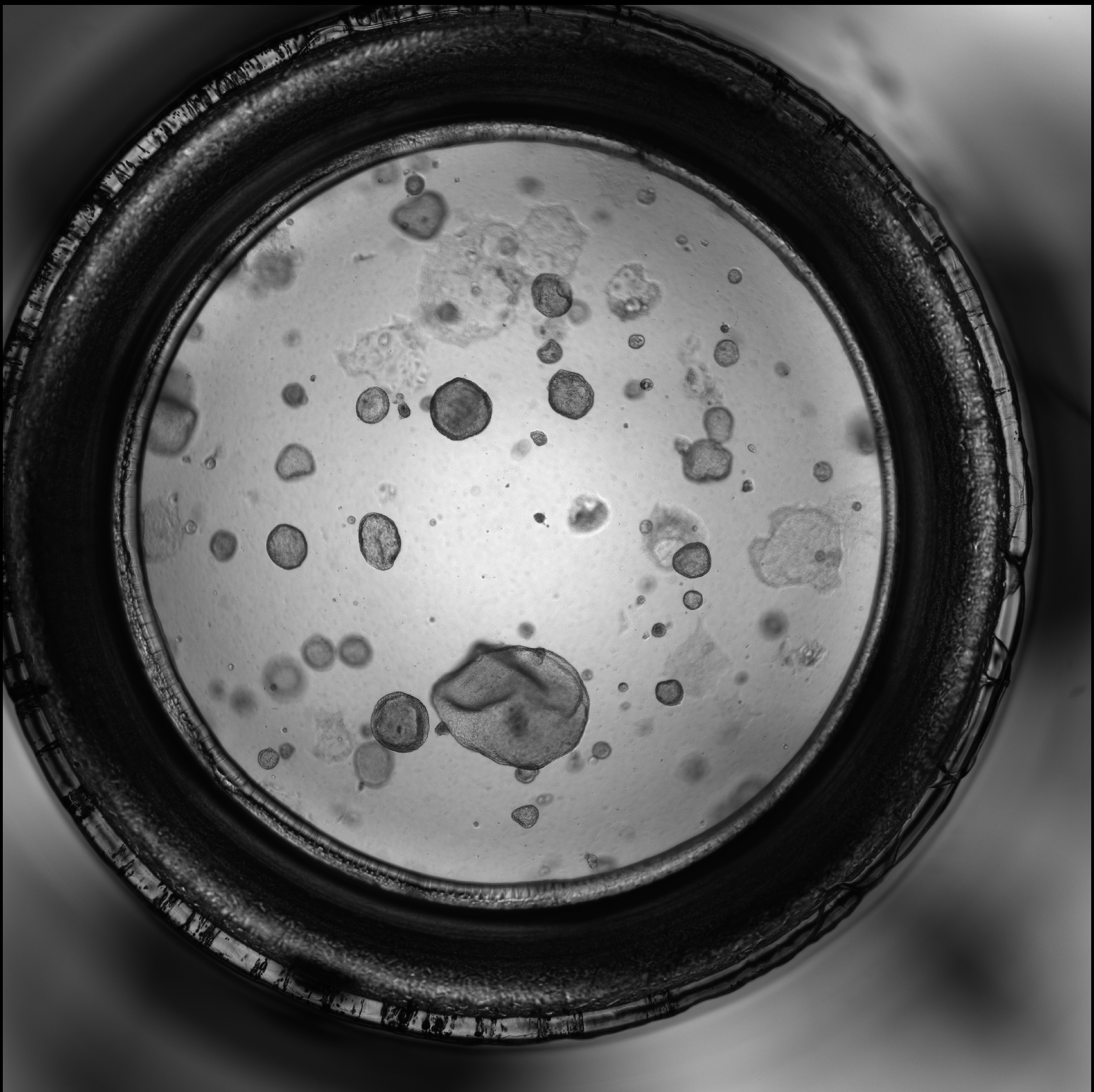

Supplement: Supplementary file 3 — Source data Fig. 4 [file 44318_2025_376_MOESM3_ESM.zip › 4F/cd44 low mono-culture rep2.tif]

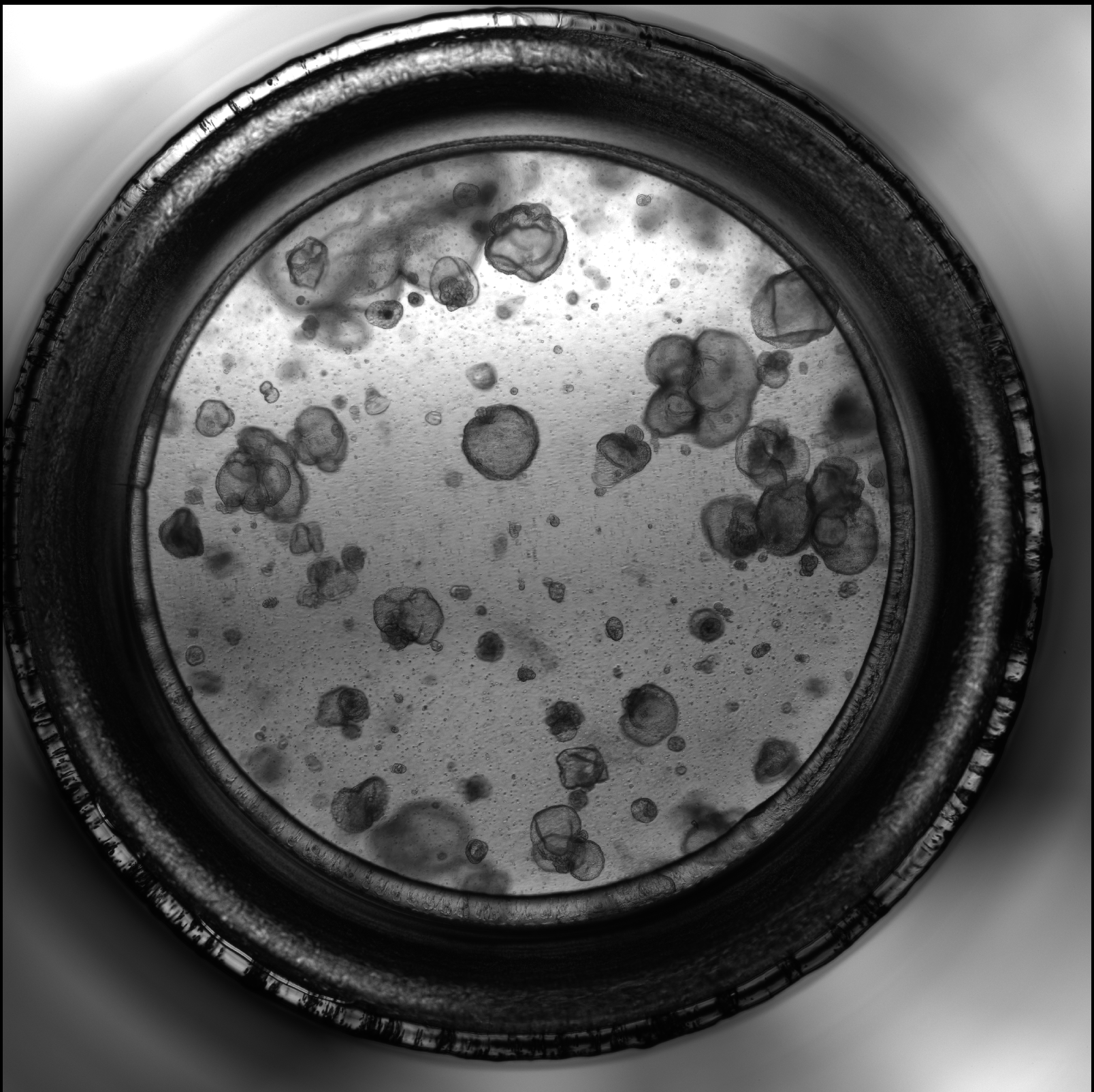

Supplement: Supplementary file 3 — Source data Fig. 4 [file 44318_2025_376_MOESM3_ESM.zip › 4F/CD44 high co-culture rep1.tif]

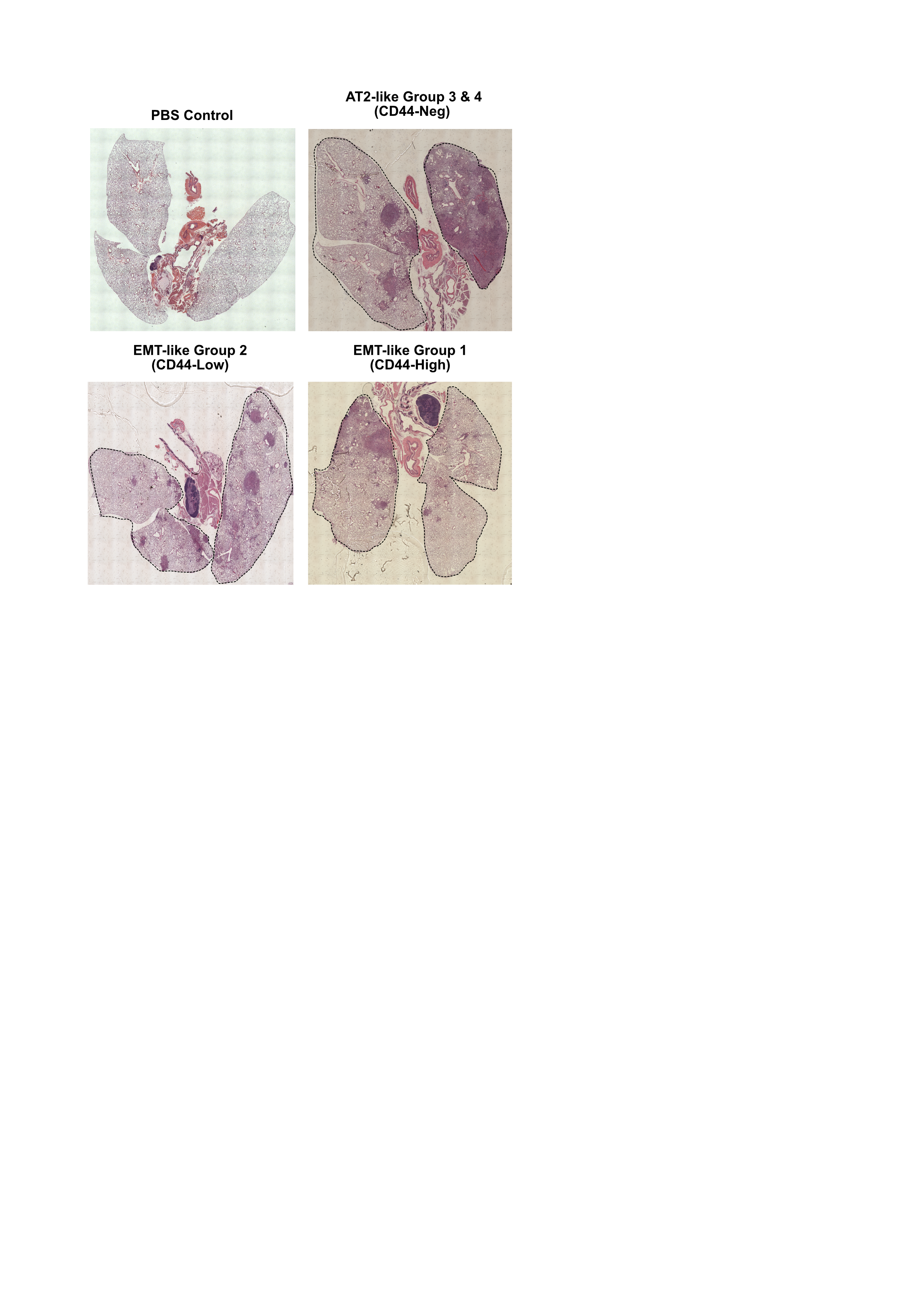

Supplement: Supplementary file 4 — Source data Fig. 5 [file 44318_2025_376_MOESM4_ESM.zip › 5C/Original large image for showing grading lesion.tiff]
