# Supplementary material for: Organoid modeling reveals the tumorigenic potential of the alveolar progenitor cell state
Source: EMBO J. 2025 Feb 10;44(6):1804–28. doi: 10.1038/s44318-025-00376-6 (PMC11914084; doi:10.1038/s44318-025-00376-6)
Supplement: Supplementary file 5 — Expanded View Figures [file 44318_2025_376_MOESM5_ESM.pdf]

Expanded View Figures

Figure EV1. related to Fig. 1. Cell state definition by combining gene expression assay and chromatin accessibility assay from one single cell.

(**A**) UMAP projection of scMulti-omic gene expression data of KPY and YFP control organoids. Cells are colored by RNA clusters. (**B**) UMAP projection of scMulti-omic gene expression data of KPY and YFP control organoids. Cells are colored by sample ID. (**C–E**) Umap plots highlighting expression level (Log(TPX + 1), color bar of Umaps and Y axis of boxplots) of selected genes. (**F**) UMAP projection of scMulti-omic gene expression data of KPY organoids. Cells are colored by RNA clusters. (**G**) UMAP projection of scMulti-omic chromatin accessibility data of KPY organoids. Cells are colored by cell clusters identified in Fig. EV1F. (**H**) Each cluster identified from scMulti-omic gene expression data (Fig. EV1F) is illustrated in Chromatin accessibility UMAP. (**I**) Heatmap showing the highly expressed genes in each group of cells. (**J**) Umap plots highlighting expression level (Log(TPX + 1), color bar of Umaps and Y axis of boxplots) of selected genes.

 © The Author(s)

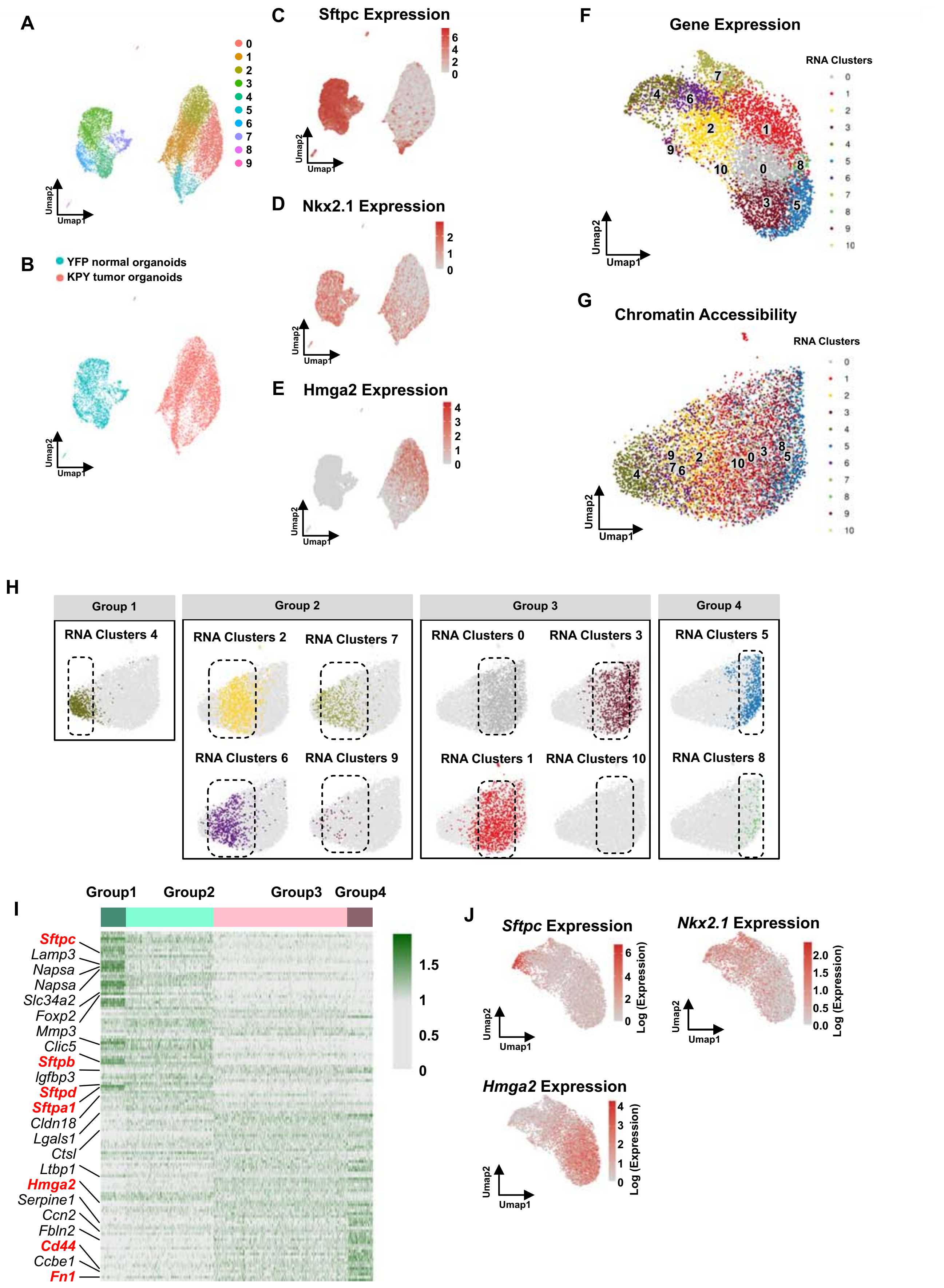
A
B
YFP normal organoids
KPY tumor organoids
C
Sftpc Expression
D
Nkx2.1 Expression
E
Hmga2 Expression
F
Gene Expression
RNA Clusters
G
Chromatin Accessibility
RNA Clusters
Umap1
Umap2
H
Group 1
Group 2
Group 3
Group 4
RNA Clusters 4
RNA Clusters 2
RNA Clusters 7
RNA Clusters 0
RNA Clusters 3
RNA Clusters 5
RNA Clusters 6
RNA Clusters 9
RNA Clusters 1
RNA Clusters 10
RNA Clusters 8
I
Group1
Group2
Group3
Group4
Sftpc
Lamp3
Napsa
Napsa
Slc34a2
Foxp2
Mmp3
Clic5
Sftpb
Igfbp3
Sftpd
Sftpa1
Cldn18
Lgals1
Ctsl
Ltbp1
Hmga2
Serpine1
Ccn2
Fbln2
Cd44
Ccbe1
Fn1
J
Sftpc Expression
Nkx2.1 Expression
Hmga2 Expression
Log (Expression)

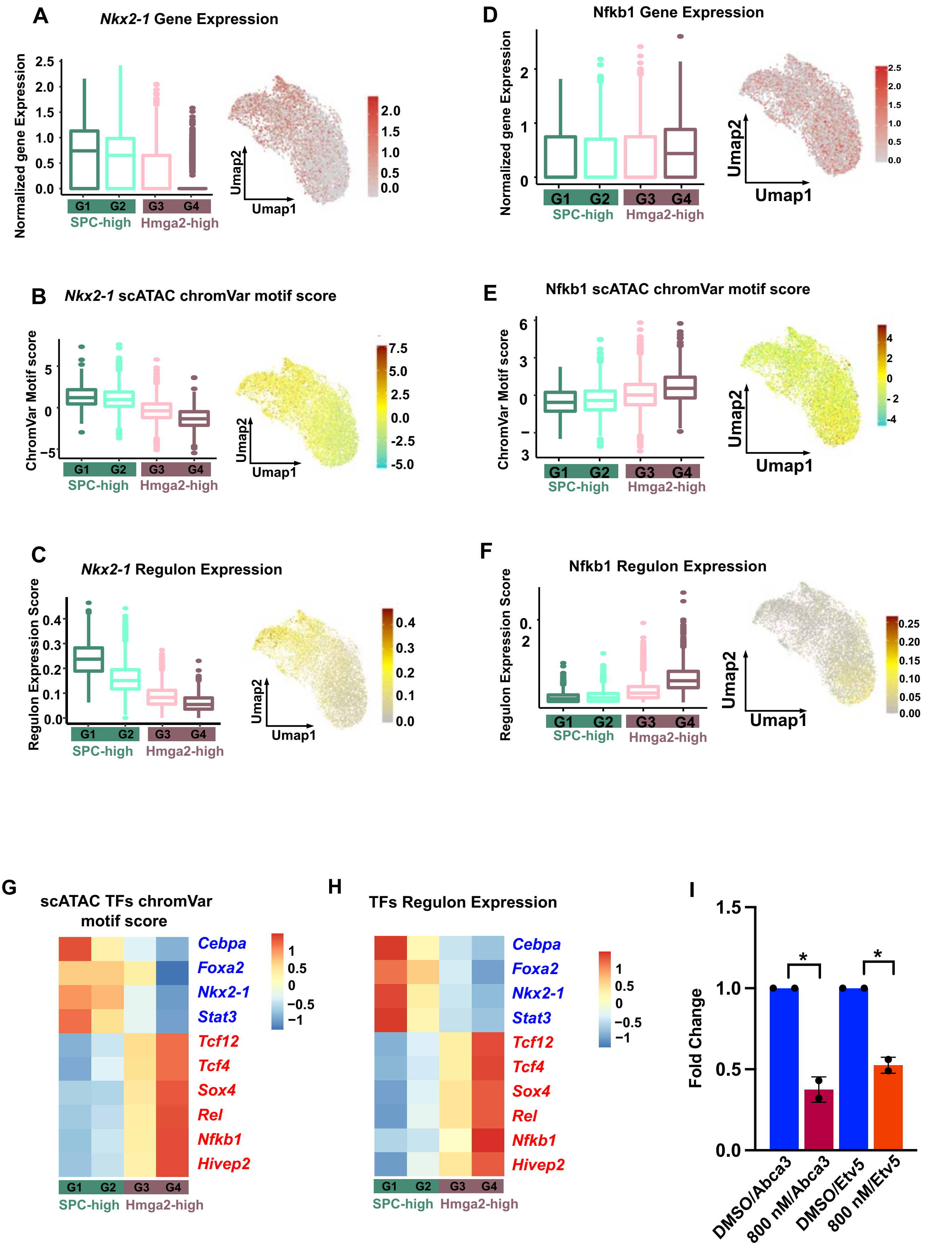
A
Nkx2-1 Gene Expression
Normalized gene Expression
G1 G2 G3 G4
SPC-high Hmga2-high
Umap1
Umap2
D
Nfkb1 Gene Expression
B
Nkx2-1 scATAC chromVar motif score
ChromVar Motif score
E
Nfkb1 scATAC chromVar motif score
C
Nkx2-1 Regulon Expression
Regulon Expression Score
F
Nfkb1 Regulon Expression
G
scATAC TFs chromVar motif score
Cebpa
Foxa2
Nkx2-1
Stat3
Tcf12
Tcf4
Sox4
Rel
Nfkb1
Hivep2
H
TFs Regulon Expression
I
Fold Change
DMSO/Abca3
800 nM/Abca3
DMSO/Etv5
800 nM/Etv5

 © The Author(s)

Figure EV2. related to Fig. 2.

(**A–C**) Showing candidate regulator for SPC-high cells. The gene expression level (**A**), motif enrichment score (**B**) and Regulon expression score (**C**) of Nkx2.1 are shown both in boxplots and Umap. The central line represents the median, the box encompasses the interquartile range (IQR) (25th to 75th percentile), and the whiskers extend to the minimum and maximum values within 1.5 times the IQR. Outliers are shown as individual points beyond the whiskers. G1: $n = 773$; G2: $n = 2514$; G3: $n = 4367$; G4: $n = 769$. (**D–F**) Showing candidate regulator for Hmga2-high cells. The gene expression level (**D**), motif enrichment score (**E**), and regulon expression score (**F**) of Nfkb1 are shown both in boxplots and Umap. The central line represents the median, the box encompasses the interquartile range (IQR) (25th to 75th percentile), and the whiskers extend to the minimum and maximum values within 1.5 times the IQR. Outliers are shown as individual points beyond the whiskers. G1: $n = 773$; G2: $n = 2514$; G3: $n = 4367$; G4: $n = 769$. (**G**, **H**) Summary of all candidate regulators for SPC-high and Hmga2-high cells. The motif enrichment score (**G**) and regulon expression score (**H**) for each candidate regulator are shown using heatmaps. (**I**) qPCR showing that Stattic treatment (800 nM) on day 7 KPY organoid cells reduced the expression of putative Stat3 target genes Abca3 and Etv5, compared to DMSO treatment. The data represents the mean ± SD ($n = 2$). *P*-value was calculated using an unpaired t-test with Welch's correction. *p*-Values = 0.035 and 0.046 (from left to right).

© The Author(s)

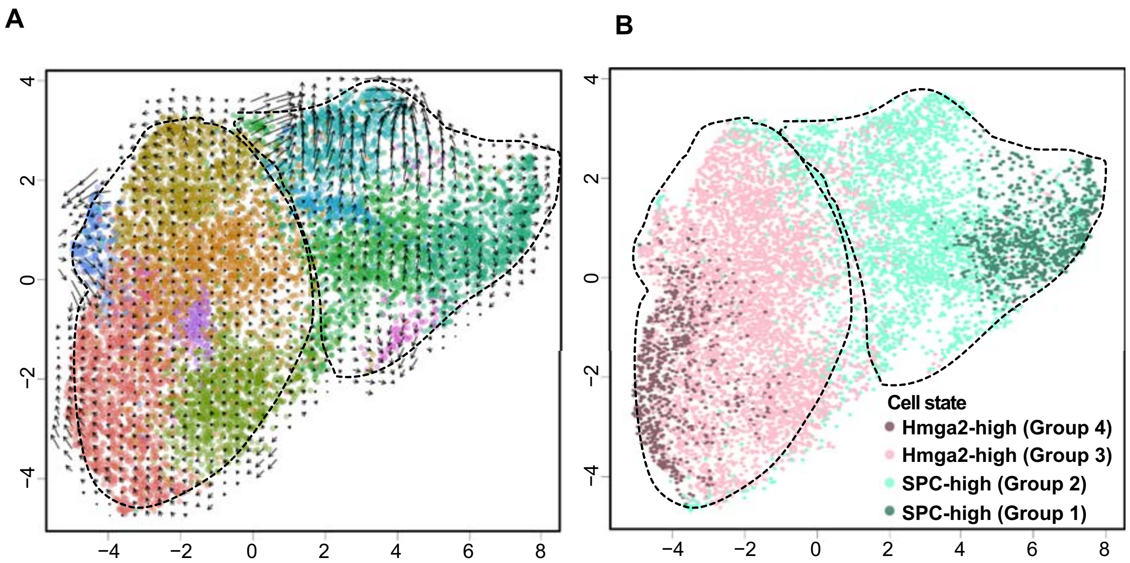

Figure EV3. related to Fig. 3. Pseudotime analysis reconstructs tumorigenesis trajectory in tumor organoids.

(**A**) RNA velocity analysis of 7 day KPY tumor organoids. (**B**) Cell states identity are plotted on the RNA velocity UMAP.

 © The Author(s)

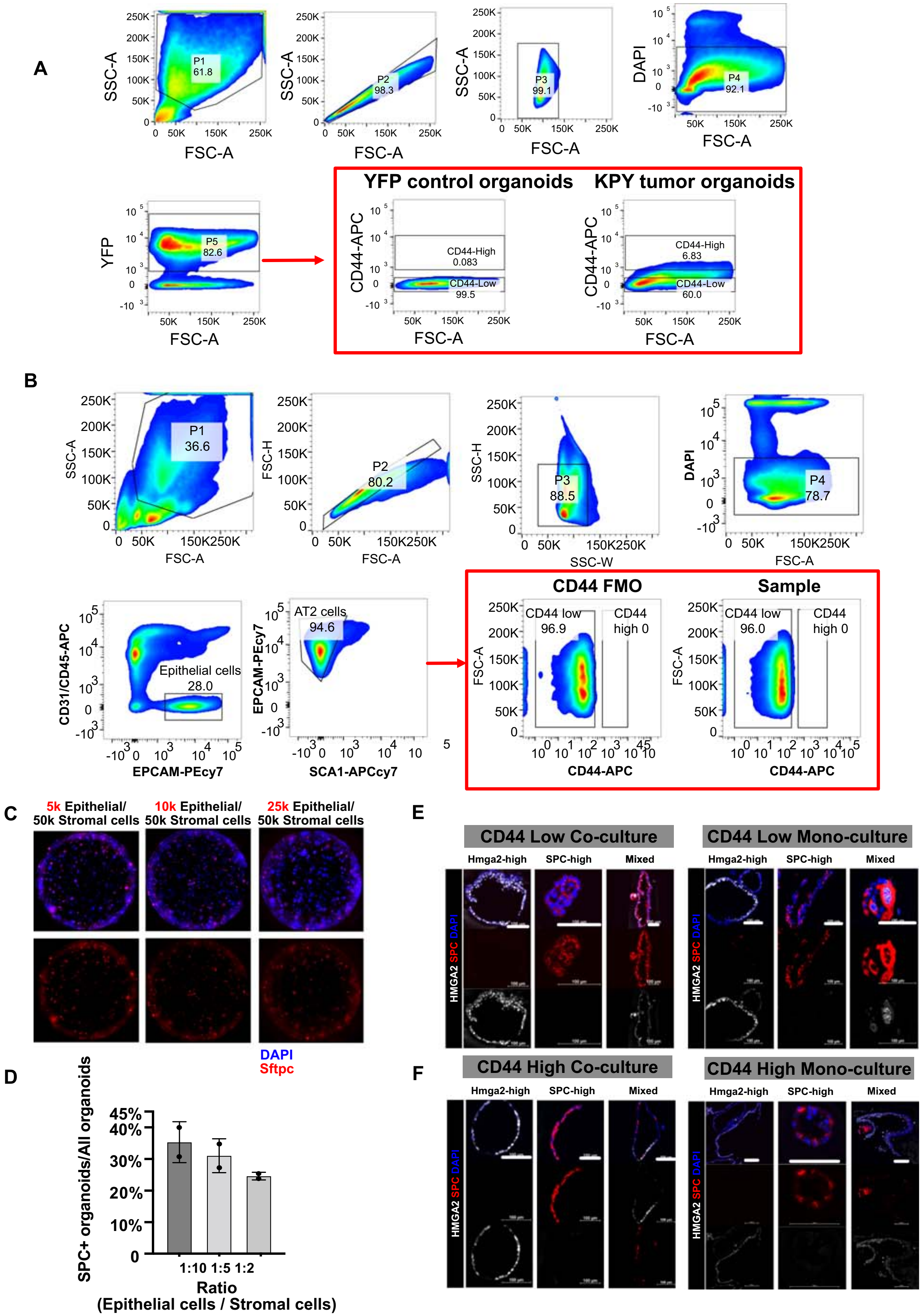
A
B
C
D
E
F
YFP control organoids
KPY tumor organoids
CD44 FMO
Sample
5k Epithelial/ 50k Stromal cells
10k Epithelial/ 50k Stromal cells
25k Epithelial/ 50k Stromal cells
DAPI
Sftpc
SPC+ organoids/All organoids
Ratio
(Epithelial cells / Stromal cells)
CD44 Low Co-culture
CD44 Low Mono-culture
CD44 High Co-culture
CD44 High Mono-culture
Hmga2-high
SPC-high
Mixed

Figure EV4. related to Fig. 4. Co-culture with lung mesenchymal cells enhanced the organoids forming ability of SPC-high cells but not Hmga2-high cells.

(**A**) FACS strategy for subsetting two cell states from 7 days KPY organoids using CD44. (**B**) Check the expression of CD44 in freshly sorted AT2 cells (DAPI-/CD31-/CD45-/EPCAM+/SCA1-). CD44 FMO control was used to set the CD44-neg and CD44-high gate. (**C**) Representative pictures of whole mount staining on 7 days KPY organoids in different conditions when the ratio between epithelial cells and mesenchymal cells is 1:10, 1:5, 1:2. (**D**) Bar plot showing the percentage of SPC+ organoids in three conditions when the ratio between epithelial cells and mesenchymal cells is 1:10, 1:5, 1:2. The data represents the mean ± SD. (**E**) Representative pictures of 7 days SPC+/Hmga2- organoids (SPC-high), SPC-/Hmga2+ organoids (Hmga2-high) and SPC +/Hmga2+ organoids (Mixed) derived from CD44-neg population in Co-culture and Mono-culture condition. Scale bar, 100 µm. (**F**) Representative pictures of 7 days SPC+/Hmga2- organoids (SPC-high), SPC-/Hmga2+ organoids (Hmga2-high) and SPC+/Hmga2+ organoids (Mixed) derived from CD44-high population in Co-culture and Mono-culture condition. Scale bar, 100 µm.

 © The Author(s)

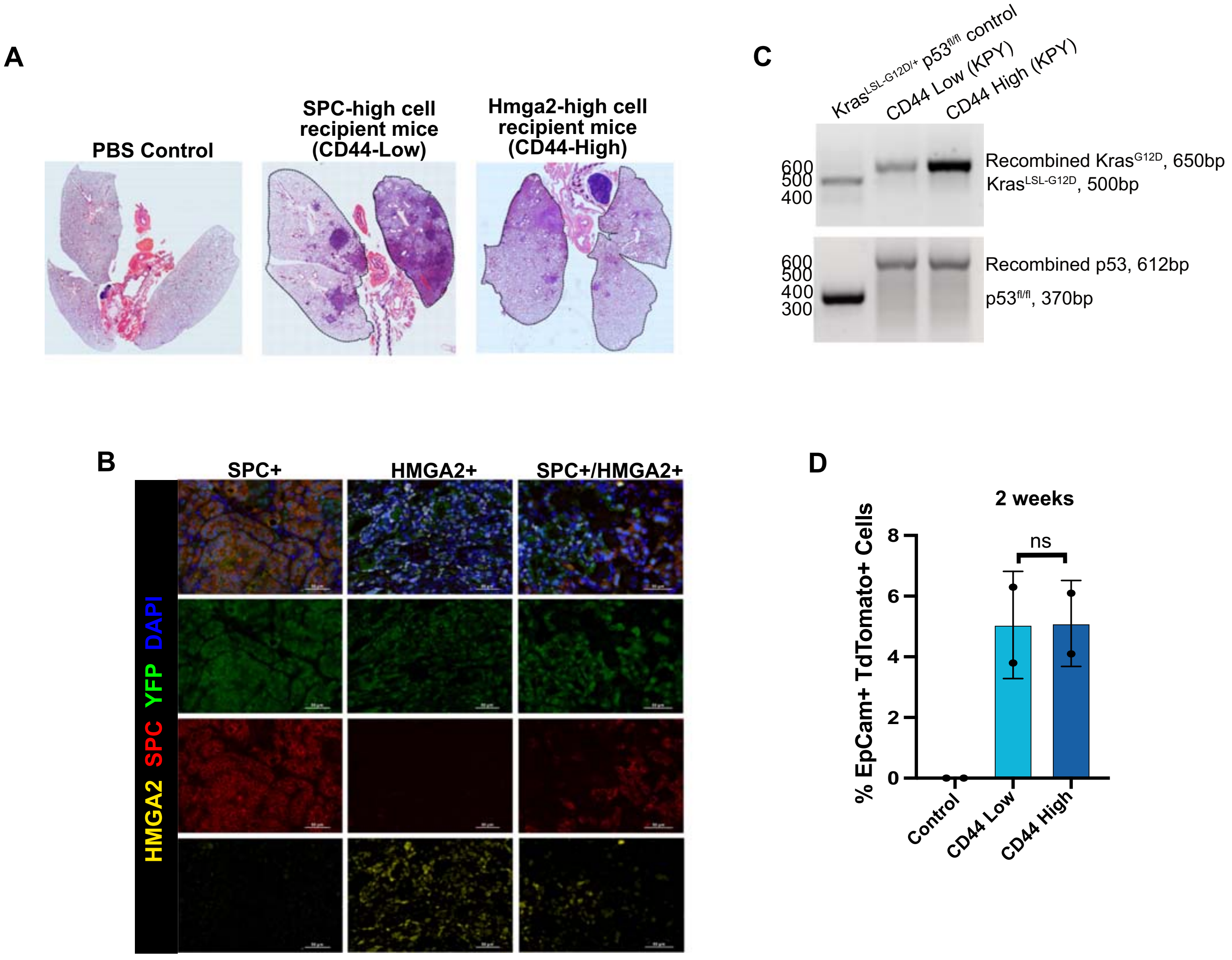

Figure EV5. related to Fig. 5. SPC-high cells have higher tumorigenic capacity than Hmga2-high cells in vivo.

(**A**) HE staining of lungs from PBS control, CD44-neg, and CD44-high recipient mice. (**B**) IF staining showing the expression of SPC and Hmga2 in lesions from tumor organoids recipient mice. Both CD44-neg recipient mice and CD44-high recipient mice can derive SPC+, HMGA2+, SPC+/HMGA2+ tumors. Scaled bar = 50 μM. (**C**) Recombination PCR data showing no amplified bands corresponding to the unrecombined Kras or p53 alleles in both the CD44-low and CD44-high KPY cell population. (**D**) Bar diagram quantifying the percentage of DAPI-/EpCam+/TdTomato+ from mice injected with CD44-low and -high organoid cells. N.s. non-significant. The data represents the mean ± SD. *p*-value was calculated using an unpaired t-test with Welch's correction. *p*-Value = 0.9780 (compare light blue vs. dark blue bars).
